# A Novel Live-Attenuated Vaccine Candidate for Mayaro Fever

**DOI:** 10.1371/journal.pntd.0002969

**Published:** 2014-08-07

**Authors:** William J. Weise, Meghan E. Hermance, Naomi Forrester, A. Paige Adams, Rose Langsjoen, Rodion Gorchakov, Eryu Wang, Maria D. H. Alcorn, Konstantin Tsetsarkin, Scott C. Weaver

**Affiliations:** Institute for Human Infections and Immunity, Center for Tropical Diseases, and Sealy Center for Vaccine Development, University of Texas Medical Branch, Galveston, Texas, United States of America; Centers for Disease Control and Prevention, United States of America

## Abstract

Mayaro virus (MAYV) is an emerging, mosquito-borne alphavirus that causes a dengue-like illness in many regions of South America, and which has the potential to urbanize. Because no specific treatment or vaccine is available for MAYV infection, we capitalized on an IRES-based approach to develop a live-attenuated MAYV vaccine candidate. Testing in infant, immunocompetent as well as interferon receptor-deficient mice demonstrated a high degree of attenuation, strong induction of neutralizing antibodies, and efficacy against lethal challenge. This vaccine strain was also unable to infect mosquito cells, a major safety feature for a live vaccine derived from a mosquito-borne virus. Further preclinical development of this vaccine candidate is warranted to protect against this important emerging disease.

## Author Summary

Mayaro virus (MAYV) is a mosquito-borne alphavirus that causes severe and sometimes chronic arthralgia in persons in South America, where it circulates in forest habitats. It is widely neglected because it is typically mistaken for dengue due to the overlap in the clinical signs and symptoms, and the lack of laboratory diagnostics in most endemic locations. Furthermore, MAYV has the potential to initiate an urban transmission cycle like that of dengue, which could result in a dramatic increase in human exposure. Because there is no effective vaccine or specific treatment, we developed a candidate vaccine to protect against MAYV infection. We used an attenuation approach based on the elimination of the MAYV subgenomic promoter and insertion of a picornavirus internal ribosome entry site to mediate translation of the structural proteins. This vaccine was well attenuated in mouse models, highly immunogenic, and protected against fatal MAYV infection. Our results indicate that this MAYV strain is promising for further development as a potential human vaccine.

## Introduction

Mayaro virus (MAYV) is an important and growing human health concern in the neotropics. First isolated in Mayaro county, Trinidad in 1954, cases of Mayaro fever (MAY) have since been reported in 9 different countries in northern South America [1]. In addition, serological surveys suggest that MAYV has expanded into the Central American countries of Costa Rica, Guatemala, and Panama [2]. Typical presentations of MAY consist of an acute febrile illness accompanied by headache, retro-orbital pain, myalgia, vomiting, diarrhea, and rash [3]. However, the hallmark manifestation of MAY is arthralgia [4], which is often severe and debilitating, and can persist for up to a year, with recurring relapses possible. The high incidence of dengue fever in the same areas in which MAYV circulates, and the similarity of the initial signs and symptoms, leads to the misdiagnosis and underreporting of MAY cases [5], [6]; therefore, MAYV is typically neglected as an important cause of tropical diseases. For example, in several areas of northern South America approximately 1% of all febrile illness that is clinically similar to dengue is caused by MAYV [7].

MAYV is a zoonotic pathogen that circulates in an enzootic cycle involving *Haemagogus spp.* mosquitoes and as yet unidentified vertebrate hosts [3]. Although seropositivity has been detected in birds and rodents, non-human primates have consistently demonstrated the highest rates of antibodies, suggesting that they are the principal reservoir hosts. Infection of humans typically occurs in communities near humid tropical forests, and is often associated with logging or other forest activities [1], [8]–[10]. However, as land use and demographic changes in South America lead to human populations expanding within regions of tropical forest, an increasingly higher percentage of the population may be at risk [11]. In addition, the demonstration that the urban mosquito, *Aedes aegypti*, can transmit MAYV after exposure to bloodmeals with titers approximating human viremia levels [5], [12] raises the concern that the virus could emerge into an urban transmission cycle similar to that of its close relative, chikungunya virus (CHIKV).

**Figure 1:**
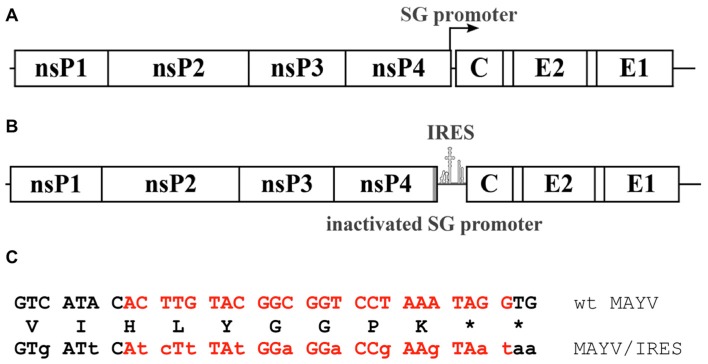
Genetic characteristics of MAYV vaccine strain. A. Wild-type genome including subgenomic promoter; B. Vaccine strain with inactivated subgenomic promoter; C. Subgenomic promoter sequence (red lettering) with synonymous mutations indicated by lower-case letters. The deduced amino acid sequence is shown between the nucleotide sequences.

MAYV belongs in the family *Togaviridae*, genus *Alphavirus*. Despite circulating exclusively in the New World, MAYV belongs genetically, antigenically [13], [14]. The genome of MAYV is a single-stranded, positive sense RNA, approximately 11.45 Kb in length that encodes 4 nonstructural proteins (nsP1-4) on the 5′ end and 3 structural proteins on the 3′ end, including the capsid and envelope glycoproteins, E1 and E2 (Fig. 1) [13], [15]. Genomic RNA includes 2 open reading frames (ORFs); the nonstructural polyprotein ORF is translated in a cap-dependent manner from genomic RNA, while the structural polyprotein ORF is translated from a subgenomic RNA transcript, which is also capped [16], [17].

There is no licensed vaccine available for MAY, and current control strategies rely on reducing human exposure to potentially infected mosquito vectors. Only one attempt to generate a vaccine for MAYV infection is described in the literature [18]. Formalin inactivation of wild-type (wt) MAYV strain TRVL15537 was tested in immunocompetent CD-1 mice using a single vaccination. This vaccine was immunogenic, and some efficacy was demonstrated via passive transfer of immune mouse sera to infant mice, followed by lethal challenge.

The ideal MAYV vaccine would produce rapid, long-term immunity after a single dose to rapidly control outbreaks, with a low risk of adverse side effects. The vaccine would also need to be cost effective for use in resource-poor parts of Latin America, and easy to produce. For a live-attenuated vaccine, which typically meets most of these criteria, mosquito-transmission incompetent would also be highly desirable for use in non-endemic locations.

To produce such a vaccine, we employed an attenuation strategy involving an encephalomyocarditis virus (EMCV) internal ribosome entry site (IRES), which has been successfully used for other alphavirus vaccines [19]–[24]. Replacement of the subgenomic promoter reduces expression of the structural proteins, which are now translated via the IRES from genomic RNA, and the inefficient recognition of the IRES by insect ribosomes results in a phenotype that is also incapable of replicating in mosquito cells [25]. For this study, we tested the efficacy of an IRES-based vaccine candidate for MAYV (henceforth called MAYV/IRES), which was highly attenuated, efficacious, and safe when tested in murine models.

## Materials and Methods

### Design and production of the MAYV/IRES vaccine candidate

A full-length genomic cDNA clone was generated from MAYV strain CH using RT-PCR and standard cloning methods as described previously [20]. The virus strain, a 2001 human isolate from Iquitos, Peru, was obtained from the World Reference Center for Emerging Viruses and Arboviruses at the University of Texas Medical Branch. It was passaged once on Vero cells before RNA extraction. Details on primers and restriction sites are available upon request.

To produce an attenuated MAYV that was capable of replicating in vertebrate cells, but not in invertebrate cells, the translation of viral structural proteins was placed under control of the EMCV IRES, directly downstream from the subgenomic promoter. The subgenomic promoter was also inactivated with 14 synonymous mutations using standard PCR-based mutagenesis methods (Fig. 1). These mutations were chosen to inactivate the promoter while preserving the amino acid sequence of the nsP4 C-terminus. A single PCR-derived amplicon containing mutated subgenomic promoter and IRES sequence was cloned into wt MAYV plasmid at SanDI – NcoI sites. The complete cDNA clone was sequenced to ensure that no errors occurred during PCR amplifications or cloning. Plasmid DNA was linearized with PacI prior to *in vitro* transcription, semi-quantified by gel electrophoresis, and recombinant viral RNA was electroporated into Vero cells using conditions described previously [20]. Titers of rescued wt MAYV and MAYV/IRES were both 4.0×10^7^ PFU/mL at 28 h post electroporation. Cell culture supernatants were harvested 28 h post electroporation, centrifuged to pellet cell debris, and stored at −80°C.

### Animals

All mice were purchased from Charles River Laboratories (Wilmington, MA). Animal studies were approved by the University of Texas Medical Branch Institutional Animal Care and Use Committee.

### Cell culture

African green monkey kidney (Vero) and human fetal lung fibroblast cells (MRC-5) cells were purchased from the American Type Culture Collection (ATCC, Manassas, VA) and maintained in culture with Dulbecco's Modified Eagle's Medium (DMEM) supplemented with 5% fetal bovine serum (FBS) and gentamicin sulfate and incubated at 37°C with 5% CO_2_. *Aedes albopictus*-derived C6/36 cells were maintained in DMEM supplemented with 10% FBS, 1% tryptose phosphate broth (TPB) solution, and an antibiotic mixture of penicillin/streptomycin at 29°C and 5% CO_2_.

### Virus replication assays

Vero and MRC-5 cells were used to assess the replication kinetics of the MAYV/IRES vaccine candidate and wt MAYV. Cells were grown to 95% confluency in 6-well plates. Virus was added to each well at a multiplicity of infection (MOI) of 0.1 plaque forming units (PFU)/cell in triplicate and incubated with the cells for 1 h. The cells were then washed twice with phosphate buffered saline (PBS) to remove residual virus, and 2 mL of medium were added to each well. At designated timepoints (6, 12, 24, 36 and 48 hours post infection (hpi) for Vero cells, and 24, 48, 72, and 96 hpi for MRC-5 cells), the culture supernatant was harvested for virus titration by plaque assay, then fresh medium (2 mL) was added to replace the volume.

### Virus passaging

To assess the stability of the MAYV/IRES vaccine candidate, 5 passages were performed in duplicate on both Vero and C6/36 *A. albopictus* cells in T25 flasks, with the cells at 95% confluency before infection at a MOI of 0.1 PFU/cell. As a control, wt MAYV was also passaged. Vero cells were incubated at 37°C and 5% CO_2_ for 48 h, while the C6/36 *Ae. albopictus* cells were incubated at 29°C and 5% CO_2_ for 72 h. Culture supernatants were then collected and used to infect a new flask at the same MOI. Virus titers from each passage were measured by plaque assay.

### Sequencing

To evaluate the genetic stability of the MAYV/IRES vaccine candidate, viral genomes from Vero passages 3 and 5 of both MAYV/IRES and wt MAYV were fully sequenced. Viral RNA was extracted using a QIAamp Viral RNA Mini Kit (Qiagen, Valencia, CA). This was followed by RT-PCR which was performed in a two-step reaction process involving SuperScript III One-Step RT-PCR System (Invitrogen, Grand Island, NY) in conjunction with Phusion High-Fidelity DNA Polymerase (New England Biolabs, Ipswich, MA). PCR amplicon sizes were confirmed by gel electrophoresis and then purified by a QIAquick PCR Purification Kit (Qiagen). A BigDye kit (Applied Biosystems, Foster City, CA) was then used to prepare the samples for Sanger sequencing. Thirty-nine overlapping amplicons were used to cover the entire genome; primer sequences are available from the authors.

### Animal studies

Infant outbred CD1 mice have been shown to develop disease similar to humans for the arthralgic alphavirus CHIKV [26], and were therefore chosen as a model to evaluate the MAYV/IRES attenuation. Cohorts of six-day-old outbred CD1 mice were infected over the dorsum subcutaneously (SC) with 10^4^ PFU, a dose used previously [26], and were subsequently monitored daily for 10 days for survival and body weight. To evaluate immunogenicity, cohorts of adult 28-day-old CD1 mice were also infected SC with 10^5^ PFU, and survival and body weights were monitored daily until day 28 post infection. Mice were bled on days 1–3 after infection, and serum was tested for viremia by plaque assay [27] to assess attenuation. On day 28 post infection, the animals were bled and a plaque reduction neutralization test (PRNT) was performed on the sera to measure antibodies as described previously [27].

MAYV produces no detectable disease in adult, immunocompetent mice. Therefore, to assess attenuation, cohorts of ca. 5–8-week-old interferon type I receptor-deficient A129 mice were infected intradermally (ID) on the left footpad (FP) with 10^4^ PFU. The animals were monitored for survival, body weight changes, and viremia. Footpad swelling was also measured using a caliper at the site of inoculation. At day 28 post infection, sera were collected and PRNTs were performed. On day 29 post infection, the mice were challenged SC with 10^4^ PFU of wt MAYV strain CH. The mice were monitored the following 7 days for survival, change in body weight, and viremia.

### Ethics statement

The University of Texas Medical Branch (UTMB) Institutional Animal Care and Use Committee approved the animal experiments described in this paper under protocol 02-09-068. UTMB complies with all applicable regulatory provisions of the U.S. Department of Agriculture (USDA) - Animal Welfare Act; the National Institutes of Health (NIH), Office of Laboratory Animal Welfare - Public Health Service (PHS) Policy on Humane Care and Use of Laboratory Animals; the U.S Government Principles for the Utilization and Care of Vertebrate Animals Used in Research, Teaching, and Testing developed by the Interagency Research Animal Committee (IRAC), and other federal statutes and state regulations relating to animal research. The animal care and use program at UTMB conducts reviews involving animals in accordance with the *Guide for the Care and Use of Laboratory Animals* (2011) published by the National Research Council.

### Statistical analysis

Analysis of variance (ANOVA) followed by a Tukey's post-hoc test, Kruskall-Wallis with Bonferroni correction for multiple comparisons, Kaplan-Meier, and Mann-Whitney test were performed using Prism 5 (GraphPad Software, La Jolla, CA). P-values<0.05 were considered significant.

## Results

### Replication kinetics of the MAYV/IRES vaccine candidate

**Figure 2:**
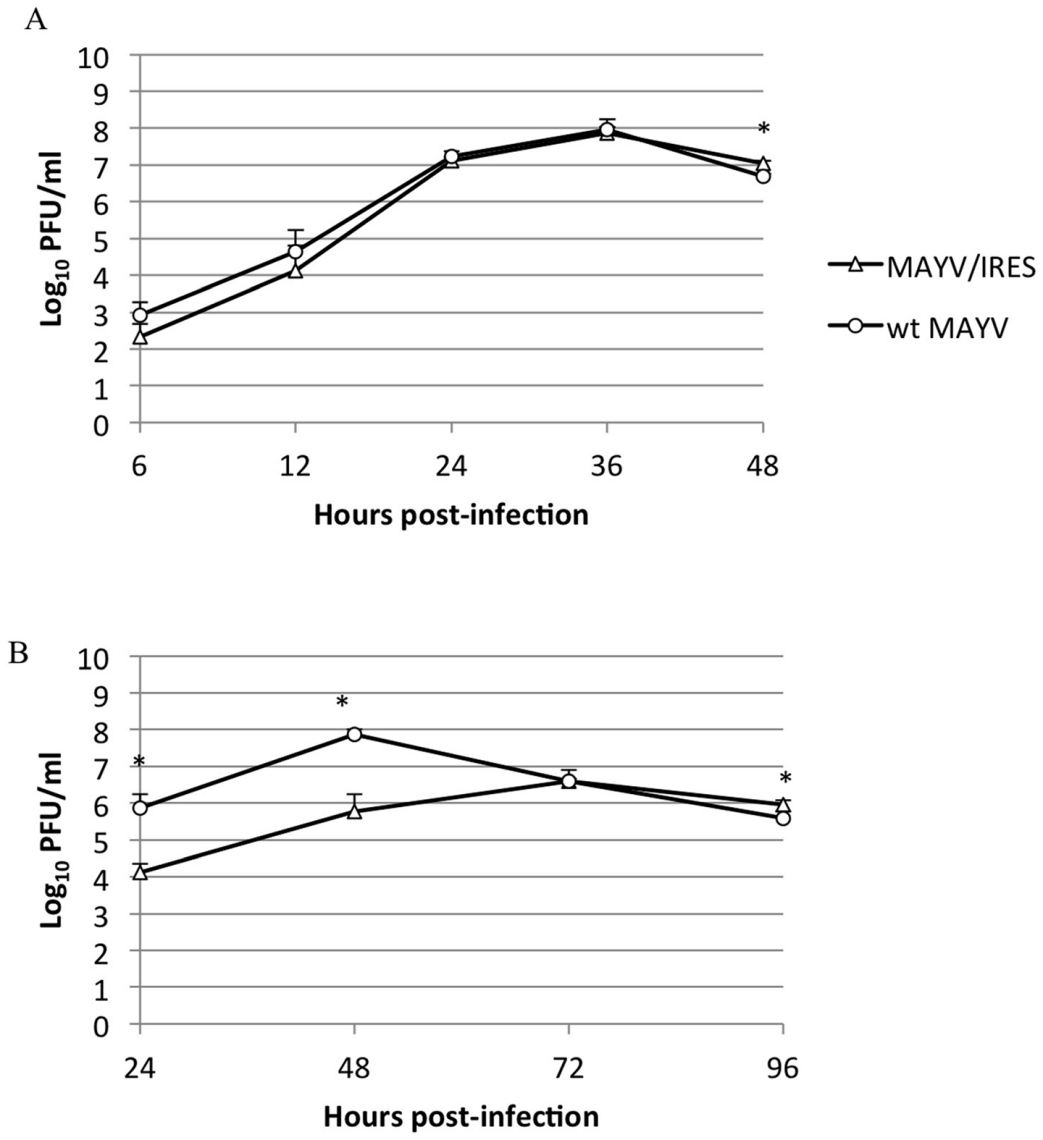
Replication kinetics of MAYV/IRES and wt MAYV after infection of A) Vero or B) MRC-5 cells at a multiplicity of infection (MOI) of 0.1 PFU/cell. n = 3. * = p<0.05. Error bars indicate standard deviations.

To assess the replication kinetics, virus derived from electroporated Vero cells was compared to wt MAYV after infection of Vero cells (Fig. 2A). Infections were performed in triplicate (n = 3) at a MOI of 0.1 PFU/cell. Both MAYV/IRES and wt MAYV titers peaked 36 hpi, but wt MAYV had a slightly higher titer of 1.1×10^8^ PFU/mL while MAYV/IRES had a peak titer of 7.8×10^7^ PFU/mL. Significant differences were seen only at the 48 hpi timepoint (ANOVA, p<0.05). Plaque morphology was consistent throughout the experiment, with wt MAYV having a slightly larger (0.5–3 mm) and more diffuse plaque morphology than MAYV/IRES (0.5–2 mm) under 0.4% agarose in MEM (48 h incubation).

MRC-5 cells are well characterized and widely used in cell culture-based vaccine production. Therefore, we also measured the replication kinetics of the MAYV/IRES vaccine candidate, as well as wt MAYV on this cell line in triplicate wells (n = 3) at a MOI of 0.1 PFU/cell (Fig. 2B). The MAYV/IRES virus reached a peak titer of 10^6.7^ PFU/ml at 72 hpi, which was much later and at a lower titer than wt MAYV. Plaque morphology measured on Vero cells of MAYV/IRES virus derived from MRC-5 or Vero cells was comparable.

### Stability of the MAYV/IRES vaccine candidate

The stability of MAYV/IRES was tested *in vitro* by 5 serial passages in Vero cells, in duplicate at an MOI of 0.1 PFU/cell. MAYV/IRES maintained a slightly lower titer than wt MAYV throughout the passages, with a range of 4.2×10^7^ PFU/mL after passage 2, to a peak of 1.9×10^8^ PFU/mL after passage 3; wt MAYV titers remained between 10^8^ and 10^9^ PFU/mL (data not shown). To evaluate the genetic stability of the MAYV/IRES vaccine candidate, the complete consensus sequences of passages 3 and 5 were determined using overlapping amplicons generated by RT-PCR, and no mutations were detected.

MAYV/IRES was also serially, blind passaged 5 times in C6/36 *A. albopictus* mosquito cells to confirm its lack of mosquito host range. As expected, the virus was not detected during any passage, while wt MAYV replicated to high titers (data not shown).

### Assessment of MAYV/IRES attenuation in Infant CD1 Mice

Cohorts of 6-day-old CD1 mice were infected SC with 10^4^ PFU of either MAYV/IRES (n = 14), wt MAYV (n = 15), or were sham-infected with PBS (n = 15). Mice infected with wt MAYV began to die starting 3 dpi and complete mortality was seen by day 8 (data not shown). All MAYV/IRES- and sham-infected mice survived until the study was terminated 10 days after inoculation. As expected, the wt MAYV-infected cohort did not gain weight as quickly as the MAYV/IRES- or sham-infected animals, and the average weight of wt-infected animals declined rapidly beginning 4 days post-infection. There was no significant difference in weight change between MAYV/IRES- and sham-infected animals (Kruskall-Wallis with Bonferroni correction for multiple comparisons).

### Assessment of MAYV/IRES in Adult CD1 Mice

**Figure 3:**
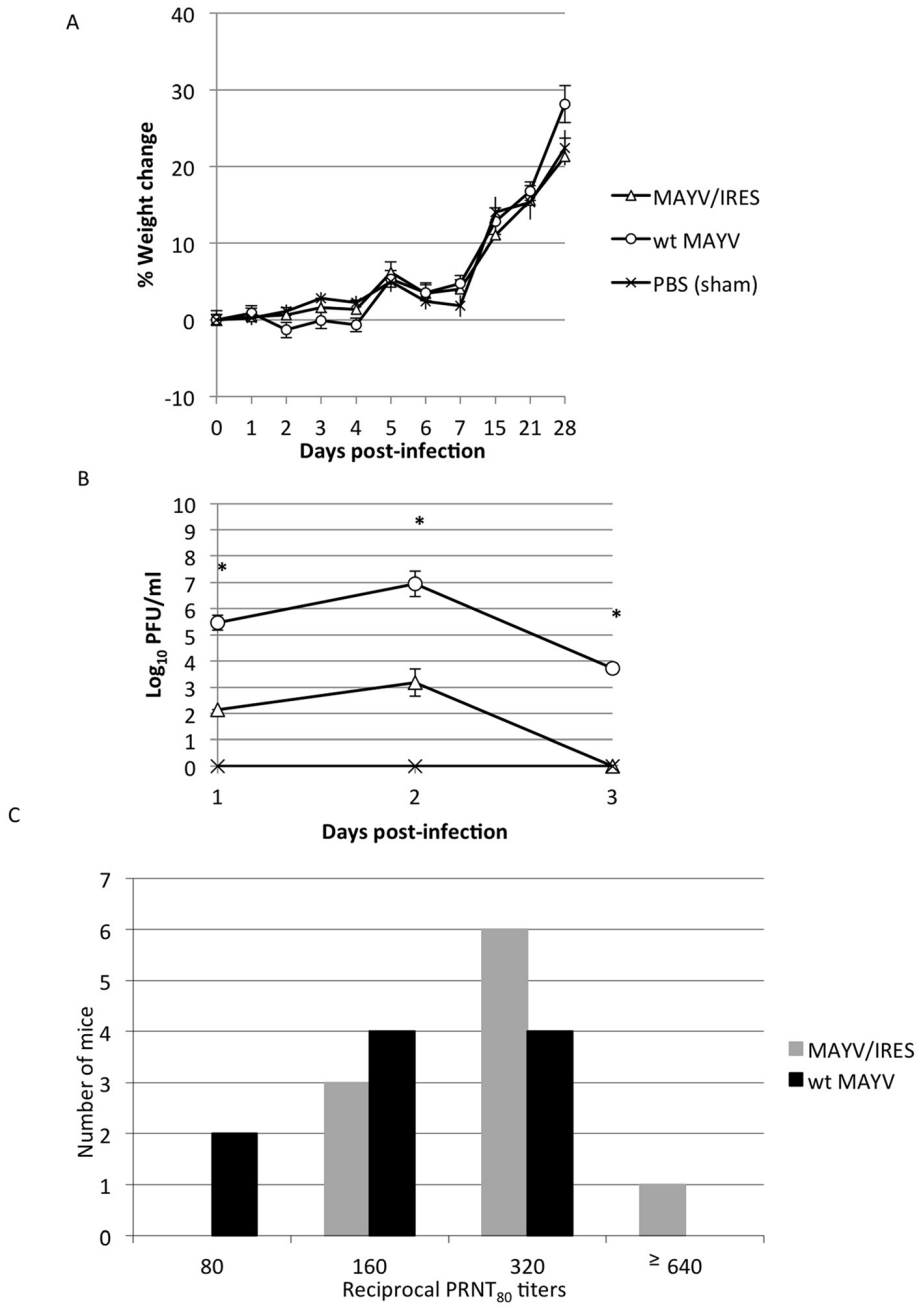
Infection of 28-day-old CD1 mice with 10^5^ PFU of MAYV/IRES or wt MAYV. A) percent change in body weight, B) viremia, and C). PRNT_80_ titers for each cohort. * = p<0.05. Error bars indicate standard deviations.

Due to the high mortality in infant CD1 mice infected with wt MAYV, adult CD1 mice (28 days-old) were also tested as a potential virulence model. Mice were infected SC with 10^5^ PFU of either MAYV/IRES (n = 10) or wt MAYV (n = 10), and negative controls were sham (PBS)-infected (n = 6). Unlike the infant 6-day-old CD1 mice, the 28-day-old mice all survived infection with wt MAYV until the study was terminated 28 days after infection. To assess with greater sensitivity signs of disease, the animals were weighed post-vaccination (Fig. 3A). The MAYV/IRES- and sham-infected cohorts gained weight steadily throughout the experiment, while the wt MAYV-infected mice lost some weight initially, but recovered by day 5 post-infection, then proceeded to gain weight in a manner similar to the other cohorts. However, these differences in weight change were not significant (p≥0.07, Kruskall-Wallis with Bonferroni correction for multiple comparisons).

To quantify viral loads of the MAYV/IRES vaccine candidate, viremia was assessed post-vaccination (Fig. 3B). Both MAYV/IRES and wt MAYV produced a peak viremia titer at day 2 post-infection, but MAYV/IRES viremia was of shorter duration and of significantly lower mean peak titer, just over 10^3^ PFU/mL, compared to 10^7^ PFU/mL for wt MAYV.

Serum neutralizing antibody titers were measured at 28 days post-infection using an 80% PRNT. MAYV/IRES titers ranged from 160 to ≥640 (mean = ≥304), and were not significantly different from those of wt MAYV-infected animals (Kruskall-Wallis with Bonferroni correction for multiple comparisons) (Fig. 3C).

### Assessment of MAYV/IRES attenuation in A129 Mice

**Figure 4:**
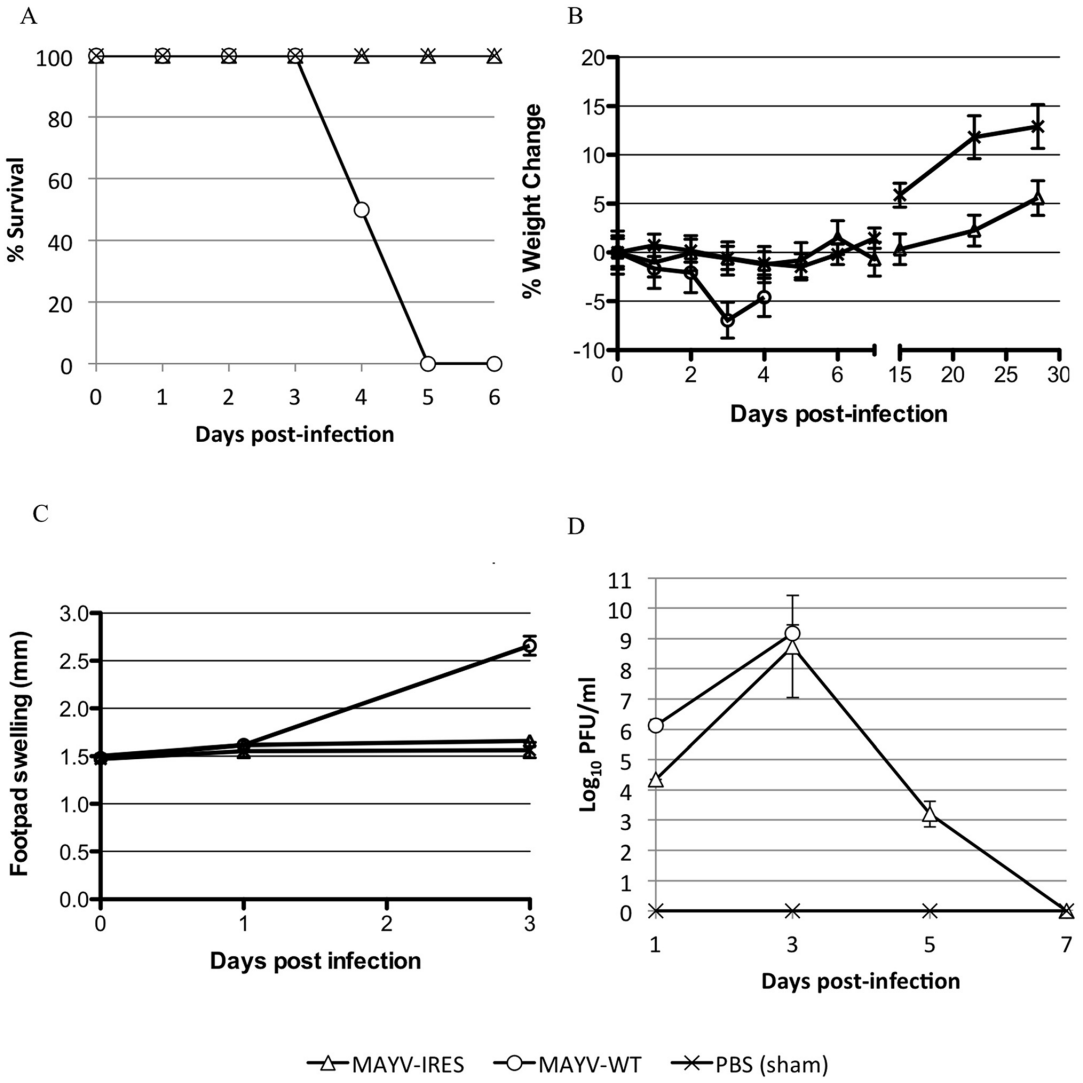
Infection of A129 mice with 10^4^ PFU of MAYV/IRES or wt MAYV. A) percent survival, B) percent change in body weight, C) Footpad swelling, and D) viremia for each cohort. * = p<0.05. Error bars indicate standard deviations.

A129 mice lack functional type 1 interferon receptors and are therefore a very sensitive model for human arthritic alphavirus infection [28]. They have been used as a lethal model for alphavirus vaccine safety and challenge studies [20]. Cohorts of adult A129 mice (n = 8) were infected with MAYV/IRES or wt MAYV, or sham-infected with PBS. Injections were performed intradermally on the left footpad with 10^4^ PFU. All MAYV/IRES- and sham-infected mice survived until the experiment was terminated on day 28, while all wt MAYV-infected mice died by day 5 (Fig. 4A). Both the MAYV/IRES and wt MAYV cohorts lost weight initially, but wt MAYV-induced loss was more dramatic and significantly greater than that of the MAYV/IRES-infected animals (Fig. 4B) (p<0.01, Kruskall-Wallis with Bonferroni correction for multiple comparisons). There was no significant difference in footpad swelling among cohorts until 3 days after infection, when wt MAYV-infected mice showed a large increase in footpad diameter, which was significantly greater than mean swelling of both MAYV/IRES- and sham-infected cohorts (Fig. 4C) (p<0.01, Kruskall-Wallis with Bonferroni correction for multiple comparisons).

Viremia was measured post-vaccination to quantify the viral load (Fig. 4D). Both MAYV/IRES and wt MAYV cohorts reached high titers in the peripheral blood, with MAYV/IRES peaking at day 3 post-infection with a titer of 5.5×10^8^ PFU/mL and wt MAYV reaching a slightly higher titer of 1.4×10^9^ PFU/mL. Differences were significant only on day one post-infection (p<0.001, Kruskall-Wallis with Bonferroni correction for multiple comparisons).

### Immunogenicity and efficacy in A129 mice

**Figure 5:**
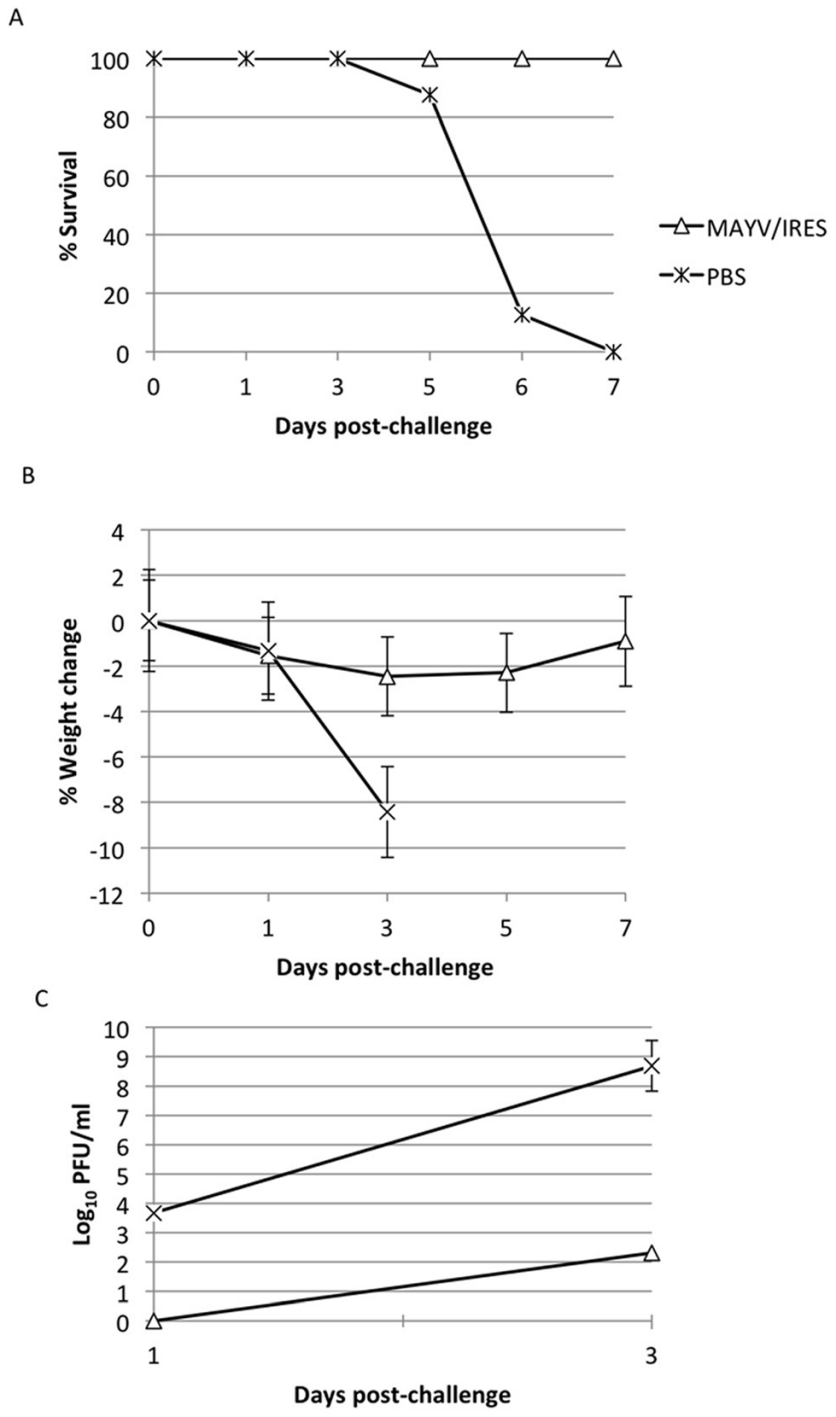
Response to challenge of MAYV/IRES-vaccinated A129 mice. A) percent survival, B) percent change in body weight, and C) viremia for each cohort following challenge. Error bars indicate standard deviations.

At day 28 post-infection, 7 of the 8 MAYV/IRES-vaccinated A129 mice had neutralizing antibody titers ≥640, while the remaining mouse had a titer of 320 (mean = ≥604). The mean PRNT antibody titer for A129 mice was significantly higher than that for CD1 immunocompetent mice (Student's T-test, p<0.01), possibly reflecting greater vaccine replication in the former (although the ages were not exactly matched). The sham-vaccinated A129 mice (n = 3) did not have detectable antibodies (<20). Mice were then challenged SC with 10^4^ PFU of wt MAYV to assess the efficacy of the MAYV/IRES vaccine. All vaccinated mice survived, while all of the sham-vaccinated mice were dead by day 7, representing a significant difference in mortality (p<0.01, Kaplan-Meier; see Fig. 5A).

To monitor disease in a more sensitive manner, weight was measured post-vaccination (Fig. 5B). The sham-vaccinated, challenged cohort lost weight more quickly and dramatically than the MAYV/IRES-vaccinated group (p<0.01, Mann-Whitney). To assess viral load, viremia post-challenge was also measured (Fig. 5C). The MAYV/IRES-vaccinated group showed a decreased viremic response upon challenge compared to the sham-vaccinated animals, only reaching a mean titer of 2.0×10^2^ PFU/mL at day 3 post-challenge, while the control group reached a much higher titer of 4.8×10^8^ PFU/mL 3 days post-challenge (p<0.05, Mann-Whitney).

## Discussion

It has been over 60 years since the discovery of MAYV in Trinidad, and there is still no licensed vaccine available despite continued outbreaks, and the potential for urban transmission in a dengue-like cycle [5], [12] that could expose millions of people. Our MAYV/IRES vaccine was designed to offer single-dose, rapid protection to protect people both in endemic regions and in the event of an urban outbreak. Previous attempts to generate a vaccine to protect against MAY focused on inactivated wt virus [18]. A single vaccination proved immunogenic in adult CD1 mice, and efficacy was demonstrated indirectly via passive transfer of the immune mouse sera to infant mice, followed by lethal challenge. However, no further testing of this vaccine has been reported.

To capitalize on the advantages of live-attenuated vaccines, including rapid and long-lasting immunity as well as ease of manufacture, we used the IRES-based attenuation approach that has been demonstrated to offer highly stable and predictable attenuation for alphaviruses [19]–[24]. Unlike traditional alphavirus attenuation derived from cell culture passages that typically relies on unstable point mutations, resulting in reactogenicity and the potential for reversion to wt virulence and transmissibility [29]–[32] the IRES-based rationale approach suppresses structural viral protein expression by elimination of the subgenomic promoter using multiple inactivating mutations. Thus, reversion is highly unlikely because the promoter sequence is very specific and intolerant of change [33], resulting in superior attenuation stability following serial mouse passages compared to traditional point mutation-dependent attenuation [22]. Further safety is achieved through the use of the encephalomyocarditis virus IRES, which inefficiently mediates translation in insect cells [25], and thus eliminates the possibility for mosquito transmission. Finally, the titers of nearly 10^8^ PFU/cell of MAYV/IRES produced by vaccine substrate-approved Vero cells should be adequate for large-scale manufacture, and the stability we demonstrated following Vero cell passages will be critical for licensure.

Like previous studies using the IRES-based alphavirus attenuation approach, our results showed that MAYV/IRES is stable in cells of mammalian origin (Vero), but incapable of efficient replication in a C6/36 *A. albopictus* cell line. Previous studies have showed that other IRES-based attenuated alphaviruses are also incapable of replication after intrathoracic inoculation into *A. albopictus* mosquitos [20], [22]. In every murine model we tested, MAYV/IRES was highly attenuated, only producing minimal signs of disease in the highly stringent A129 model that cannot mount an effective interferon response. This vaccine candidate was also highly immunogenic, inducing high levels of neutralizing antibody titers in both adult CD1 and A129 mice at 28 days post-vaccination. Challenge of A129 vaccinated mice at 29 days post-infection with a high dose of wt MAYV showed complete protection from detectable disease, despite the high virulence and complete lethality of MAYV in unvaccinated animals. These murine studies indicate that MAYV/IRES is highly attenuated, highly immunogenic, and provides strong protection against MAYV challenge. Further studies in another animal model are needed. Typically, nonhuman primates such as macaques reproduce human-like disease after alphavirus infection [24], [34]–[39]. These animals should be evaluated as models for human MAYV to determine if they will be useful for the next steps in preclinical evaluation of MAYV/IRES.

A variety of alternative vaccine development approaches are available for alphaviruses including inactivated virus, subunit protein, DNA and virus-like particles (VLP) as well as traditionally attenuated and chimeric vaccines [40], [41]. All of these approaches emphasize safety but have significant drawbacks including a multiple dose requirement for efficacy, short-lived immunogenicity necessitating boosters, challenging delivery (DNA via electroporation) and complex, expensive manufacture (VLPs) and the risk of residual live virus after inactivation, which was shown to result in the death of an eastern equine encephalitis-vaccinated horse in California [42]. Our MAYV/IRES candidate overcomes all of these shortcomings to generate rapid immunity following a single dose, and should have greatly reduced reactogenicity due to its robust, highly stable attenuation design. Although further testing should be done to evaluate the duration of protective immunity, other IRES-based alphavirus vaccines have generated completely protective immunity in macaques for over one year (C. Roy, S.C.W., unpublished). MAYV/IRES therefore should be ideal for inducing rapid, long-lived immunity after a single dose for use in developing countries where MAYV is endemic, as well as for a traveler's vaccine for persons visiting South America.

In summary, our MAYV/IRES vaccine candidate is highly attenuated and immunogenic, unable to infect mosquito cells, and provides protection from lethal challenge in murine models. These results indicate that further preclinical development of MAYV/IRES is justified for its evaluation as a potential human vaccine that could protect people from MAY in South America, but also on other locations if the virus spreads and urbanizes like the closely related CHIKV [5], [43]–[46]. Furthermore, MAYV/IRES should be evaluated for its ability to protect against CHIKV and Ross River viruses, other closely related alphaviruses that cause epidemics in Africa and Asia, or Australia and Oceania, respectively. CHIKV is of particular concern because in December of 2013 it invaded the Caribbean, representing the first autochthonous transmission in the Western Hemisphere [47]–[49]. This event could portend a major epidemic throughout the Americas if spread to the mainland occurs into dengue-endemic regions where both *A. aegypti* and *A. albopictus* mosquito vectors are present along with a nearly naïve human population. The latter vector is highly susceptible to Asian CHIKV strains with adaptive mutations that dramatically enhance its vectorial capacity [50]–[55], and it is unknown if similar mutations could enhance MAYV urbanization in a similar manner. An effective vaccine could greatly mitigate these risks and have a major impact on public health in South America.
